# Editorial: Systemic Regulation of Organ Homeostasis and Implications of Hormones and Immunity

**DOI:** 10.3389/fendo.2021.740835

**Published:** 2021-09-14

**Authors:** Rajakumar Anbazhagan, Raghuveer Kavarthapu, Premendu P. Mathur, Tatjana S. Kostic, Hridayesh Prakash

**Affiliations:** ^1^Eunice Kennedy Shriver National Institute of Child Health and Human Development, National Institutes of Health (NIH), Bethesda, MD, United States; ^2^Department of Biochemistry and Molecular Biology, School of Life Sciences, Pondicherry University, Pondicherry, India; ^3^Department of Biology and Ecology, Faculty of Sciences, University of Novi Sad, Novi Sad, Serbia; ^4^Amity Institute of Virology and Immunology, Noida, India

**Keywords:** neuroimmune interaction, microglia, neurotrophic factor, Leydig cells, Sertoli cells, circadian rhythm

Propagating life to the next generation is a tightly controlled, interdependent process whereby tissues and organ systems work together to promote an organism’s relative fitness and survival. Groups of organs and tissues performing related functions are organized into systems that interact and work cohesively to achieve normal physiological functions of the organism. Though in-depth research is available on each organ/tissue independently (human and other model organisms), the complex interactions that take place between cells, tissues, organs, and the organism are sometimes overlooked. Tissue-specific changes in the micromilieus have a fundamental influence on cellular/organ functions, and alterations to endocrine/ligand signaling can impact local and systemic tissue function, all of which may contribute to disease pathogenesis.

With this background in mind, the current topic explores the potential interactions between endocrine and immune systems that are important for understanding disease biology and investigating associated treatment strategies. Another major mandate of this Research Topic was to explore optimal treatment and management strategies for various disorders, such as cancer. The research and review articles in this topic address interactional and non-classical functions of hormones and immunity.

Circadian rhythms regulate the physiological processes of an organism, including its immune system. Understanding the molecular mechanisms underlying diurnal variation in hosts due to infection-mediated immune responses is warranted. In this context, Jacquelot et al. describe how neuro-immune interactions create rhythmic activity in innate lymphoid cells, and how minor disruptions lead to the development of chronic inflammation. The immune system and the neuroendocrine system communicate extensively through overlapping receptors and networks that control mechanisms of immunity in addition to regulating development and metabolism. Klein describes the immune-endocrine interactions with an emphasis on the hormones of the hypothalamus-pituitary-thyroid axis. In addition, the processes by which immune system–derived thyroid stimulating hormone (TSH) controls thyroid hormone synthesis and bone metamorphosis are also explained in the context of a novel splice variant of TSHβ, a contributing factor in the development of autoimmune thyroid disease. Likewise, Zhan et al. review the immune checkpoint inhibitors (ICI; group of drugs used for treating various types of malignant tumors) in relation to immune system reactivation, which results in the death of normal tissues and cells, eventually leading to immune-related adverse events. Zhan et al. also discuss the clinical manifestations, possible pathogenesis, and management of ICI-related thyroid dysfunction.

Marinkovic et al. delineate the influence of constant light (LL) on the maturity of the Leydig cells and their various endocrine roles. The effects of the LL are prominent in puberty with increased *Bmal1*, *Per1/2*, and *Reverba* and decreased pituitary genes encoding gonadotropic hormones (*Cga, Lhb, Fshb*). Further, serum androgens and markers of Leydig cell maturity/differentiation (*Insl3, Lhcgr*) and steroidogenesis (*Scarb1, Star, Cyp11a1, Cyp17a1*) were decreased, with increases in serum corticosterone. The authors conclude that LL slows the maturation of Leydig cells with their endocrine function, leading to the delay of reproductive development.

Khan et al. discuss how endocrine and paracrine pathways are regulated by sex hormones and growth factors that have direct control over Sertoli cell proliferation, differentiation, and maturation, and that can directly impact reproduction.

Benitez et al. discuss a neurotrophic factor and its role in activating noradrenergic and cholinergic systems in the rat ovary. Using estradiol valerate (a polycystic ovary phenotype model), their study provides evidence that the primary signal, nerve growth factor (NGF), activates both noradrenergic and cholinergic systems *in vivo*. This in turn increases both norepinephrine (through an NGF-dependent mechanism) and acetylcholine levels, the former of which through an NGF-dependent mechanism. This implies that NGF is the main regulator of dual autonomic control.

Ghafouri-Fard et al. discuss an interactional aspect of insulin-like growth factor signaling in the pathogenesis of neoplasia in relation to non-coding RNA. In addition, novel therapeutic strategies are suggested based on the modification of IGF signaling and identification of the impact of non-coding RNAs in this pathway.

Fujita and Yamashita focus on microglia, resident immune cells of the central nervous system (CNS), and their role in neural development in both normal physiological and pathological conditions. Further, they discuss the heterogeneity of microglia (functional, morphological, and regional heterogeneity across different CNS regions) and associated epigenetic changes coordinating gene expression. Mechanisms underlying spatiotemporal and functional diversity of microglia during developmental stages and other altered or diseased conditions are also discussed.

Liu and Wu assess the impact of intramuscular and vaginal regimens of progesterone on neonatal outcomes in HRT-frozen-thawed embryo transfer cycles. The study concludes that higher serum progesterone induced by intramuscular regimens did not change the live birth rate or neonatal outcomes compared to vaginal regimens.

## Conclusion and Future Perspective

The collection of articles presented in this topic introduces important work and extends our knowledge on the overlapping and interlinking pathways across organ systems with specific reference to hormones and immunity. With this work, we hope our community is encouraged to tackle the remaining, unresolved issues to advance the body of scientific research into the interactions between hormones and immunity. Identifying comparable and cohesive interactions locally and systemically will pave the way for better understanding and, ultimately, improve disease management and treatments.

## Author Contributions

All authors have contributed to the editing and writing equally. All authors contributed to the article and approved the submitted version.

## Conflict of Interest

The authors declare that the research was conducted in the absence of any commercial or financial relationships that could be construed as a potential conflict of interest.

## Publisher’s Note

All claims expressed in this article are solely those of the authors and do not necessarily represent those of their affiliated organizations, or those of the publisher, the editors and the reviewers. Any product that may be evaluated in this article, or claim that may be made by its manufacturer, is not guaranteed or endorsed by the publisher.

